# Analysis of Gingival Fibroblasts Behaviour in the Presence of 3D-Printed versus Milled Methacrylate-Based Dental Resins—Do We Have a Winner?

**DOI:** 10.3390/jfb15060147

**Published:** 2024-05-28

**Authors:** Veaceslav Saramet, Miruna S. Stan, Alexandra Ripszky Totan, Ana Maria Cristina Țâncu, Bianca Voicu-Balasea, Dan Sebastian Enasescu, Florentina Rus-Hrincu, Marina Imre

**Affiliations:** 1 Department of Complete Denture, Faculty of Dental Medicine, “Carol Davila” University of Medicine and Pharmacy, 020021 Bucharest, Romania; veaceslav@saramet.eu (V.S.); marina.imre@umfcd.ro (M.I.); 2 Department of Biochemistry and Molecular Biology, Faculty of Biology, University of Bucharest, 91–95 Splaiul Independentei, 050095 Bucharest, Romania; 3 Department of Biochemistry, Faculty of Dental Medicine, “Carol Davila” University of Medicine and Pharmacy, 020021 Bucharest, Romania; alexandra.ripszky@umfcd.ro (A.R.T.); dan.enasescu@drd.umfcd.ro (D.S.E.); florentina.rus-hrincu@umfcd.ro (F.R.-H.); 4 The Interdisciplinary Center for Dental Research and Development, Faculty of Dental Medicine, “Carol Davila” University of Medicine and Pharmacy, 17–23 Plevnei Street, 020021 Bucharest, Romania; biancavoicu2106@gmail.com

**Keywords:** methacrylate-based dental resins, computer-aided manufacturing (CAD/CAM), additive manufacturing (3D printing), autophagy, biocompatibility

## Abstract

Computer-aided design and computer-aided manufacturing (CAD/CAM) techniques are based on either subtractive (milling prefabricated blocks) or additive (3D printing) methods, and both are used for obtaining dentistry materials. Our in vitro study aimed to investigate the behavior of human gingival fibroblasts exposed to methacrylate (MA)-based CAD/CAM milled samples in comparison with that of MA-based 3D-printed samples to better elucidate the mechanisms of cell adaptability and survival. The proliferation of human gingival fibroblasts was measured after 2 and 24 h of incubation in the presence of these samples using a 3-(4,5-dimethylthiazol-2-yl)-2,5-diphenyltetrazolium bromide assay, and the membrane integrity was assessed through the lactate dehydrogenase release. The level of reactive oxygen species, expression of autophagy-related protein LC3B-I, and detection of GSH and caspase 3/7 were evaluated by fluorescence staining. The MMP-2 levels were measured using a Milliplex MAP kit. The incubation with MA-based 3D-printed samples significantly reduced the viability, by 16% and 28% from control after 2 and 24 h, respectively. There was a 25% and 55% decrease in the GSH level from control after 24 h of incubation with the CAD/CAM milled and 3D-printed samples, respectively. In addition, higher levels of LC3B-I and MMP-2 were obtained after 24 h of incubation with the MA-based 3D samples compared to the CAD/CAM milled ones. Therefore, our results outline that the MA-CAD/CAM milled samples displayed good biocompatibility during 24-h exposure, while MA-3D resins are proper for short-term utilization (less than 24 h).

## 1. Introduction

Computer-aided design and computer-aided manufacturing technology (CAD/CAM) includes both the subtractive (consisting of prefabricated material blocks milling or grinding) and the additive (3D printing) techniques. The subtractive technique entered the dental material industry stage in the 1980s. Since then, dental materials processing by milling techniques have been extensively studied, proving to be superior to the conventional fabrication processes in terms of their biocompatibility, and physical and mechanical properties [1,2,3]. However, additive manufacturing (3D printing) is attracting more and more attention in dentistry [4]. The fabrication of occlusal splints, crowns, and dentures represents only some of the multiple possible applications of 3D printing in the field of dentistry [5,6]. 3D printing represents a new and challenging technology, which utilizes liquid monomers in contrast to industrially prepared milling blocks [5,6]. Dental materials developed for this new and promising technique impose a special composition, but there is little information available regarding these materials’ biocompatibility [1,2,3]. Steinmassl et al. [7] revealed that dental splints obtained by the subtractive method contained comparable levels of residual unreacted monomers to those obtained from conventional manufacturing methods. However, recent studies have shown that, surprisingly, 3D-printed dental splints cured by light, in multiple-step processes, contained higher levels of residual monomers [8,9,10]. Consequently, the idea that 3D-printed oral splint materials may be more cytotoxic than milled ones can be outlined, but scientific evidence is scarce so far.

Dental restorative materials are based on combinations of different methacrylate monomers [11]. Over time, the improved methacrylate-based polymers have shown advantages, such as appreciated physical and mechanical properties and high aesthetics, while having relatively low production costs. The dental methacrylate-based materials are fabricated by the polymerization of (co-)monomers and should be considered safe. However, concerning methacrylate-based dental materials, the main unfavorable effect is the incomplete conversion of monomers to polymer chains [12,13]. Practically, ideal polymerization conditions are very hard to achieve. The polymerization degree can be controlled, mainly by parameters like the monomer-to-polymer ratio, polymerization reaction temperature, and time [12,13]. In the case of photo-polymerization, the curing time and light density play important roles [12,13]. The incomplete conversion of monomers to polymer chains triggers the release of unreacted residual (co)-monomers, reaction additives, and secondary reaction products [14,15]. Furthermore, a certain percentage of unreacted, potentially toxic monomers may remain enclosed within the polymer matrix, therefore being initially non-extractable during post-polymerization stages [16]. Moreover, in time, mechanically induced degradation processes, and chemical interaction with saliva and oral bacterial enzymes, may lead to an increased elution of the unreacted (co)-monomers from the polymer network [17,18,19,20,21,22].

Many studies have reported toxic effects triggered by unreacted monomers in dental resin. However, the cellular mechanisms of these phenomena are not yet fully elucidated. Reactive oxygen species (ROSs) are important players within the intracellular signal transduction pathways regulation [23]. ROS-regulated signaling pathways critically control inflammatory reactions, cell survival, and different cell death mechanisms. Recently, it has been shown that ROSs can upregulate autophagy as a mechanism of cell survival or death [23]. Consequently, studying these molecular signal-transduction pathways in the context of unreacted monomer-induced toxic effects represents an important step towards continuously improving the biocompatibility of methacrylate-based dental materials. 

Biocompatibility is regarded as “the ability of a material to perform with an appropriate host response in a specific application” [24]; however, this definition remains the center of an ongoing debate [25,26,27]. According to ISO standard 10993-5:2009, the direct-contact method used for viability evaluation establishes a cytotoxic potential only for values below 70% of control [28].

While there are many studies reporting data for the conventional and prefabricated milling of dental materials, very few data have been published regarding additively processed materials applicable in dentistry [29,30]. Nowadays, the most frequent clinical use of the methacrylate (MA)-based CAD/CAM milled samples and MA-based 3D-printed samples is represented by surgical guides and oral obturator prosthesis, occlusal splints, orthodontic retainers, mouthguards for sports, and diagnostic wax-ups [29,30]. However, in the case of 3D-printed materials, the commercially available resins may be used in dental clinical settings for provisional restorations and under limited periods of time, certain conditions, and strictly following the manufacturers protocols. Therefore, our in vitro study aimed to comparatively investigate the behavior of human gingival fibroblasts (HGF cell line) exposed to MA-based CAD/CAM milled samples and MA-based 3D-printed samples, in order to better elucidate the mechanisms of cell adaptability and survival. In this way, we aimed to evaluate the possible involvement of autophagy, throughout the levels of LC3B-I and matrix metalloproteinase 2 (MMP-2), as a possible adaptative and protective mechanism against cell death and as an extracellular matrix remodeling marker, respectively. Gingival fibroblasts have been selected within this study because they are the predominant cell type in the gingival connective tissue, making them highly relevant to the oral environment. By using HGF-1 cells, a direct evaluation of how these materials interact with cells found in the gingival tissue is made possible, being of significant clinical relevance as the behavior of gingival fibroblasts can directly impact the success or failure of dental restorations.

## 2. Materials and Methods

### 2.1. Additive Manufacturing (3D Printing)

The 25 samples were printed with SHERAeco-print 30 resin using the Digital Light Processing (DLP) technique. The resin was layered with a thickness of 50 μm. The angulation of 20° to the building platform was set. According to the manufacturer’s indications, after the printing procedure, the excess material and the supporting structure were been removed. Then, the printed sample was washed twice in an ultrasonic bath for three minutes. The excess and dissolved material were blown off using compressed air. The post-polymerization step consisted of two rounds of 2000 flashes of light using SHERAflashlight plus, under a constant flow of inert gas (nitrogen, 1 bar) and in the complete absence of oxygen. The characteristics, manufacturer, and lot number of the materials used are presented in Table 1.

### 2.2. Subtracting Manufacturing

**Figure 1:**
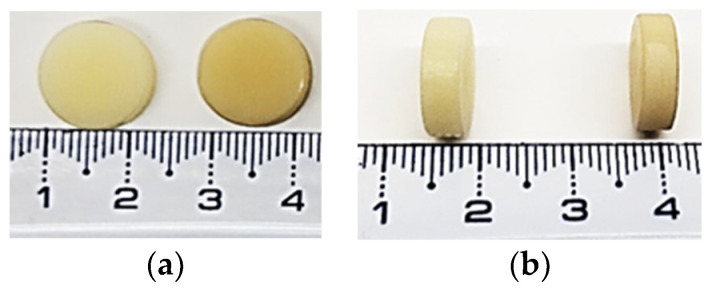
Appearance of the MA-based samples tested within this study. The size in diameter (**a**) and thickness (**b**) for the MA-based CAD/CAM (left disc in each photo) and MA-based 3D-printed (right disc in each photo) specimens used within the study.

The 25 discoidal samples were designed using CAD/CAM software, Millbox V2022-11-04. Discs with a diameter of 12 ± 2 mm and thickness of 3 ± 1 mm (Figure 1) were milled from industrially prefabricated disc PM20 blanks using the CORiTEC 350i innovative machine, manufactured by IMES I-CORE GMBH D-36132, Elterfeld, Germany.

### 2.3. Specimens Finalization

The circular surface of the fabricated discs was high-gloss polished according to a set polishing protocol (Abraso Star Glaze, a high-luster polishing paste—Bredent, Senden, Germany and a cotton cloth fine disc, Polirapid—Nordwest Dental GmbHCo. KG, Munster, Germany have been used). This meant grinding with corundum sandpaper, rubberizing with 3-stage polishers for acrylics, pro-polishing with a synthetic brush, and pumice powder. Afterwards, the specimens were cleaned in a steam jet and ultrasonic bath.

### 2.4. Cell Culture

The HGF-1 cell line (catalog no. CRL-2014) of human gingival fibroblasts, purchased from American Type Culture Collection (ATCC, Manassas, VA, USA), was cultured in complete Dulbecco’s Modified Eagle’s Medium (DMEM) with fetal bovine serum (at a final concentration of 10%) at 37 °C in a humidified atmosphere with 5% CO_2_. The detachment of cells was performed by incubation with a solution of 0.25% trypsin and 0.53 mM EDTA. The fibroblasts were seeded in 24-well plates at a cell density of 10^4^ cells/cm^2^ and left to adhere overnight. Then, the cells were incubated for the next 2 and 24 h in the presence of the two types of dental material samples: MA-based CAD/CAM and MA-based 3D-printed. The dental material samples were previously sterilized under UV light for 3 h on each side. Control samples were also used and were represented by cells unexposed to dental materials. At the end of each incubation time, the dental material samples were removed from wells and the cells were examined on an Olympus IX71 (Olympus, Tokyo, Japan) inverted fluorescence microscope. The short incubation periods were selected based on some possible clinical applications of these two types of MA-based dental materials: surgical guides and oral obturator prosthesis, occlusal splints, orthodontic retainers, mouthguards for sports, and diagnostic wax-ups.

### 2.5. Cell Viability Assay

A 3-(4,5-dimethylthiazol-2-yl)-2,5-diphenyltetrazolium bromide (MTT, Sigma-Aldrich, St. Louis, MA, USA) assay has been used for the measurement of cell viability. The growth medium was removed after 2 and 24 h of incubation, and the cells were then treated with 1 mg/mL MTT for 4 h at 37 °C. Then, 2-propanol (Sigma-Aldrich, St. Louis, MO, USA) was used to dissolve the purple formazan crystals generated in the viable cells, which were then measured for absorbance at 595 nm using a FlexStation 3 multi-mode microplate reader from Molecular Devices (San Jose, CA, USA).

### 2.6. Griess Assay

Griess reagent was prepared as a stoichiometric solution of 0.1% naphthyl ethylenediamine dihydrochloride and 1% sulphanilamide in 5% H_3_PO_4_ in order to measure the nitric oxide (NO) content in the collected culture medium after 2 and 24 h of incubation. The level of this molecule was increased after inflammation and apoptosis were initiated as a result of cytotoxic effects. Molecular Devices’ FlexStation 3 multi-mode microplate reader (San Jose, CA, USA) was utilized for recording the absorbance of the mixture made up of Griess reagent and cell culture medium at 550 nm.

### 2.7. Lactate Dehydrogenase (LDH) Assay

The culture medium collected after 2 and 24 h of cell growth in the presence of dental material samples was utilized to measure LDH release using a commercial kit (TOX7, Sigma-Aldrich, St. Louis, MO, USA) as directed by the manufacturer. In brief, 50 µL of culture supernatants were mixed with 100 µL of mix (equal parts dye, substrate, and cofactor) and incubated in the dark for 30 min. To stop the reaction, 15 µL of 1 N HCl was added, the absorbance was measured at 490 nm with a FlexStation 3 multi-mode micro-plate reader from Molecular Devices (San Jose, CA, USA), and the results were compared to the control.

### 2.8. Fluorescence Staining Assays

At the end of the incubation with dental materials, the cells were washed with PBS and incubated for 30 min at 37 °C, 5% CO_2_, with different fluorescent dyes. The calcein-AM and ethidium homodimer-1 from LIVE/DEAD™ Viability/Cytotoxicity Kit (ThermoFischer, Waltham, MA, USA) were selected to label the live and dead cells, respectively, following the indications of the kit. A concentration of 5 µM of BioTracker NucView 488 green caspase-3 dye (Sigma-Aldrich, St. Louis, MO, USA) was used to measure the intracellular activity of caspase 3/7 on a micro-plate reader set at 488 nm excitation and 520 nm emission cut-off. The amount of ROSs was quantified after labelling with 10 µM of 2′,7′-dichlorodihydrofluorescein diacetate (DCFDA, Sigma-Aldrich, St. Louis, MO, USA). For GSH detection, 5 µM of CellTracker Green 5-chloromethylfluorescein diacetate (CMFDA, ThermoFischer, Waltham, MA, USA) was used, with detection at λex/em of 492/517 nm [31]. The images were captured on an Olympus IX81 inverted fluorescence microscope, and the fluorescence intensity readings were recorded on a FlexStation 3 multi-mode microplate reader from Molecular Devices (San Jose, CA, USA). The results were compared to the control.

The Premo TM Autophagy Sensors LC3B-GFP kit (ThermoFischer Scientific, Eugene, OR, USA, catalogue number P36235) containing BacMam LC3B-GFP reagent was used to label the LC3B associated with the double-membrane of autophagosome during the autophagy process. The BacMam reagent was added to human gingival fibroblasts in suspension at the time of plating as the manufacturer indicated. The fibroblasts were maintained overnight for adhesion at 37 °C, 5% CO_2_. Then, the cells were incubated for another 24 h with the MA-based dental materials. Cells were washed with PBS and visualized on Euromex iScope microscope (Euromex Microscopen bv, Arnhem, The Netherlands).

The fluorescence intensity of the obtained images was quantified using the ImageJ version 1.54a software. The results were compared to the control, represented by cells that were not exposed to MA-based samples.

### 2.9. MMP-2 Assay

MMP-2 levels in the cell culture medium samples were measured using the Milliplex MAP kit from Millipore (Darmstadt, Germany) on an automatic analyzer Luminex 200 (Luminex Corporation, Austin, TX, USA).

### 2.10. Statistical Analysis

Data were subjected to statistical evaluation with IBM SPSS Statistics 25 and represented with the help of Microsoft Office Excel 2016. Quantitative variables were evaluated by Shapiro–Wilk test for their normal distribution. All data had a normal distribution and results were calculated as averages with standard deviations or medians with interquartile ranges. Analysis between groups was performed using Student’s/Welch’s *t*-tests and the correlations were estimated by Pearson correlation coefficients. The *p*-values less than 0.05 were considered statistically significant.

## 3. Results

### 3.1. Cell Viability Analysis

**Figure 2:**
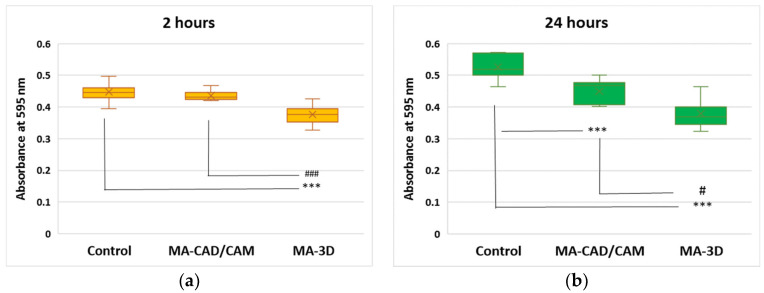
Effect of MA-based samples on cell viability. MTT assay was performed at 595 nm after 2 (**a**) and 24 (**b**) hours of human gingival fibroblasts’ incubation in the presence of MA-based CAD/CAM and MA-based 3D-printed samples. Results are presented as mean ± SD (*n* = 13). *** *p* < 0.001 compared to control, and # *p* < 0.05 and ### *p* < 0.001 compared to MA-based CAD/CAM samples.

The cell viability was assessed according to an MTT-based assay. It was observed that the MA-based CAD/CAM samples did not affect the level of cell viability after 2 h of incubation but decreased it by 14% after 24 h compared to the control (the cells without exposure to materials) (Figure 2). In contrast, the incubation with MA-based 3D-printed samples significantly reduced the viability, by 16% and 28% from control after 2 and 24 h, respectively, suggesting cytotoxicity induced to the human gingival fibroblasts (Figure 2).

**Figure 3:**
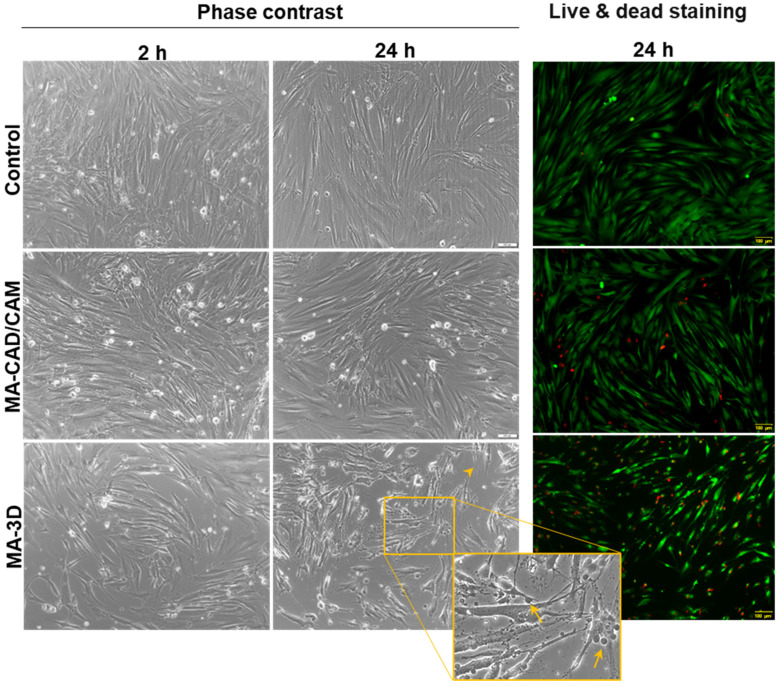
Effect on cell morphology after exposure to MA-based samples. Phase-contrast images of human gingival fibroblasts incubated for 2 and 24 h in the presence of MA-based CAD/CAM and MA-based 3D-printed samples and fluorescence staining of live (stained by calcein-AM in green) and dead (stained by ethidium homodimer-1 in red) cells (scale bar is the same for all images: 100 µm). Note the apoptotic features of fibroblasts incubated for 24 h with MA-based 3D samples (membrane blebbing indicated by arrows and cell shrinkage indicated by arrowhead). Scale bar: 100 µm.

The MA-based samples were added in the 24-well plates on top of the cells which were already attached on the plate’s surface. After 2 or 24 h, the materials were removed from the wells, and the cell morphology was analyzed by phase-contrast microscopy (Figure 3). Short periods of incubation were selected for this kind of approach to cell biocompatibility testing, as these MA-based samples are not intended to be used as orthodontic implants. Therefore, we did not check the cell attachment or proliferation on the material’s surface, which should involve longer periods of exposure. The images confirmed the presence of healthy cells in the case of the MA-based CAD/CAM samples, with a 90–95% coverage of the flask’s surface, similar to the control, confirming the good biocompatibility of this type of sample. Also, a decrease in the cell population was shown after 24-h exposure to 3D-printed samples, this finding being in accordance with the results obtained by the MTT assay. In addition, it was observed that the apoptosis of these gingival fibroblasts displayed characteristic morphological features that included cell shrinking, membrane blebbing, and apoptotic bodies formation (Figure 3), as described previously [32].

**Figure 4:**
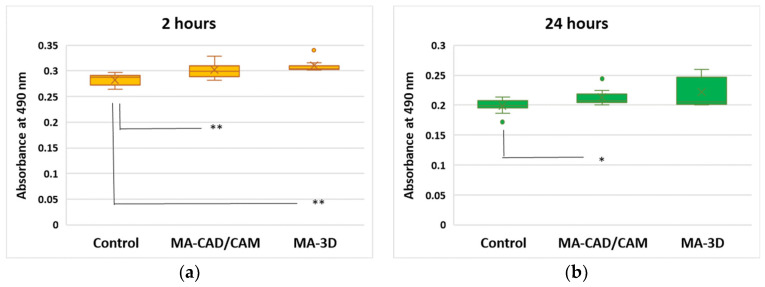
Effect of cell membrane integrity after exposure to MA-based samples. LDH release was recorded at 490 nm after 2 (**a**) and 24 (**b**) hours of human gingival fibroblasts’ incubation in the presence of MA-based CAD/CAM and MA-based 3D-printed samples. Results are presented as mean ± SD (*n* = 13). * *p* < 0.05 and ** *p* < 0.01 compared to control.

In the case of LDH release (Figure 4), a slight increase was noticed after 2 h for both samples compared to control (*p* < 0.01). Further, the highest increase was measured after 24 h of incubation with the 3D-printed sample, these results being in accordance with the cell viability decrease.

**Figure 5:**
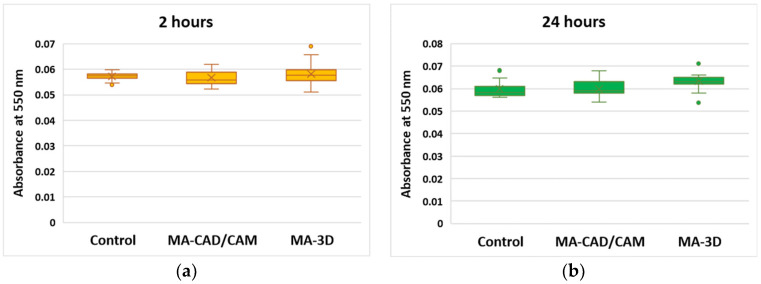
Cell toxicity after exposure to MA-based samples. NO level was measured at 550 nm after 2 (**a**) and 24 (**b**) hours of human gingival fibroblasts’ incubation in the presence of MA-based CAD/CAM and MA-based 3D-printed samples. Results are presented as mean ± SD (*n* = 13).

Regarding the NO level after the exposure to the tested samples, only a slight increase after 24 h was noticed for both types of MA samples, CAD/CAM and 3D-printed, compared to the control (Figure 5).

### 3.2. Oxidative Stress, Apoptosis and Autophagy Evaluation

**Figure 6:**
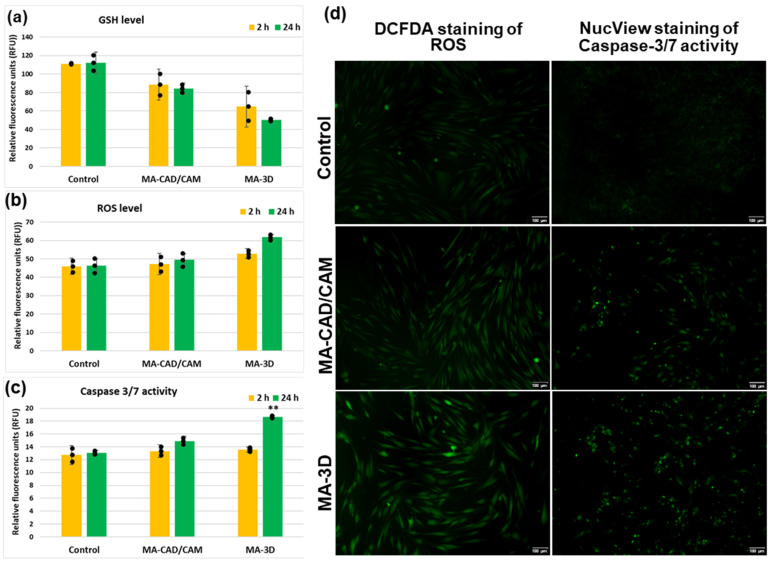
Analysis of oxidative stress and apoptosis after exposure to MA-based samples. Levels of GSH (**a**), ROS (**b**), and caspase 3/7 activity (**c**) were measured after 2 and 24 h of human gingival fibroblasts’ incubation in the presence of MA-based CAD/CAM and MA-based 3D-printed samples. Representative images of DCFDA and NucView staining in green of ROS and caspase 3/7 activity, respectively, are shown (**d**). Results are calculated as mean ± SD (*n* = 3) and replicates are represented as black dots. ** *p* < 0.01 compared to control. Scale bar: 100 µm.

In order to evaluate if oxidative stress was induced by the MA-based samples, the levels of GSH and ROSs were measured (Figure 6). There was a 25% and 55% decrease in the GSH level from control after 24 h of incubation with CAD/CAM and 3D-printed samples, respectively (Figure 6a). These results correlated very well with the ROS generation, represented by an increase of 33% above control after the same period of exposure to the MA-based 3D sample (Figure 6b), suggesting that oxidative stress was initiated in the gingival fibroblasts. Furthermore, a significantly elevated activity of caspase 3/7 (142% of control) was recorded for the 3D-printed sample after 24 h (Figure 6c), showing that the oxidative stress was accompanied by the apoptosis of fibroblasts.

**Figure 7:**
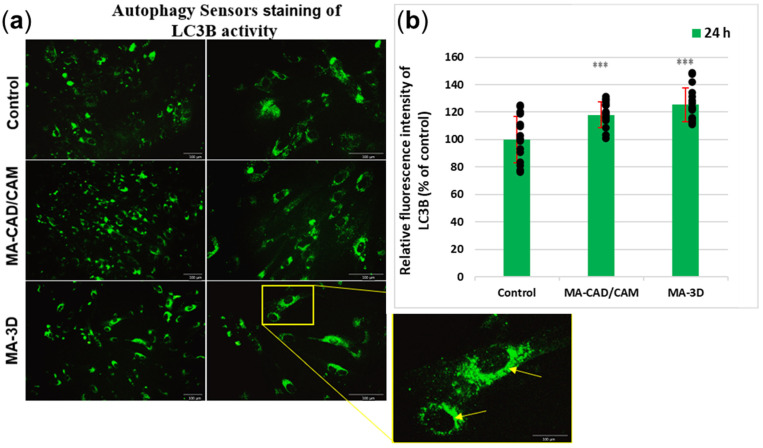
Analysis of autophagy after exposure to MA-based samples. (**a**) Representative images of Autophagy Sensors staining in green of LC3B expression are shown after 24 h of fibroblasts’ incubation with MA-based 3D samples. Note the autophagic features of fibroblasts (perinuclear-formed autophagosomes associated with LC3B protein indicated by yellow arrows). Scale bar: 100 µm. (**b**) The fluorescence intensity of LC3B expression was quantified after 24 h of human gingival fibroblasts’ incubation in the presence of MA-based CAD/CAM and MA-based 3D-printed samples. Results are calculated as mean ± SD (*n* = 15) and expressed relative to control (unexposed cells). Replicates are represented as black dots. *** *p* < 0.001 compared to control.

In the case of the cells incubated with MA-based CAD-CAM samples for 24 h, our results (Figure 7) have shown significant differences for LC3B (*p* > 0.001) when compared with control cells. The fluorescence intensity increased in the case of both samples compared to the control; however, a slightly higher degree of autophagy induction was noticed for the MA-3D samples. The images obtained with a higher magnification (40×) highlighted the perinuclear localization of autophagosomes (yellow arrows in Figure 7a (sample MA-based 3D-printed)).

### 3.3. Analysis of MMP-2 Expression

**Figure 8:**
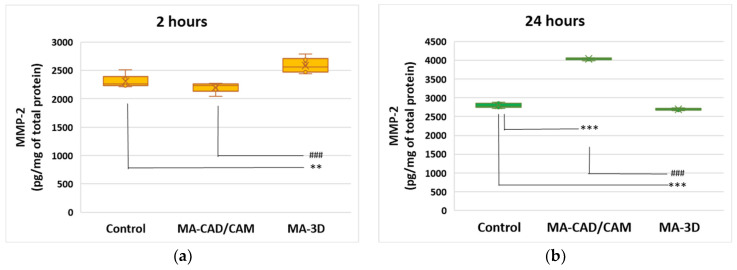
Effect of MA-based samples on MMP-2 expression. Levels of MMP-2 (**a**,**b**) were measured after 2 and 24 h of human gingival fibroblasts’ incubation in the presence of MA-based CAD/CAM and MA-based 3D-printed samples. Results are presented as mean ± SD (*n* = 3). ** *p* < 0.01 and *** *p* < 0.001 compared to control, and ### *p* < 0.001 compared to MA-based CAD/CAM samples.

Taking into account the oxidative stress-induced changes the extracellular matrix was evaluated through the level of MMP-2. The data shown in Figure 8 illustrate no major differences in MMP-2 between the cells incubated with MA-based 3D-printed samples for 24 h and the control cells. However, our findings revealed that the MMP-2 levels were significantly increased (*p* < 0.001) in the cells exposed to MA-based CAD-CAM compared to control.

## 4. Discussion

Only a small number of studies have focused so far on the potential cytotoxicity of fast-developing 3D-printed dental materials. In the case of MA-based polymers, especially those obtained by additive techniques, the MA monomers, released from the polymer matrix due to incomplete polymerization and/or resin degradation, are the main factor responsible for the major biological negative effects [27,33,34]. Taking into account these facts, our study aimed to comparatively observe the behavior of human gingival fibroblasts exposed to MA-based CAD/CAM milled samples and MA-based 3D-printed samples (both of them being mostly used for occlusal splints, orthodontic retainers, and mouthguards), in order to better elucidate the cellular mechanisms of adaptability and survival.

Our results revealed that the MA-based CAD/CAM samples did not affect the level of cell viability after 2 h of incubation, but slightly decreased the number of viable cells after 24 h compared to the control (Figure 2 and Figure 3), correlating with the preserved membrane integrity revealed by no LDH release above control (Figure 4). Regarding the NO level after the exposure to the MA-based CAD/CAM samples, it was a difference was not noticed after 24 h compared to the control (Figure 5). As it was shown that NO is a potent modulator of homeostasis through the prevention or induction of apoptosis [35], this can explain our experimental findings, namely the good cell viability in the presence of MA-based CAD/CAM samples. Similar results were reported by Xia Wei et al. [36]. Their findings revealed that no sign of apoptosis or necrosis was detected in the case of CAD/CAM dental polymeric materials [36]. Surprisingly, the incubation of gingival fibroblasts with MA-3D samples significantly reduced the viability compared with the control cells-induced membrane damage (Figure 2, Figure 3 and Figure 4), suggesting a cytotoxic effect induced by the MA-based 3D samples.

In order to understand the molecular mechanisms underlying the fibroblasts’ behavior in the presence of these two types of dental materials, we have further aimed to explore the autophagic pathway, which might play the role of a possible adaptive mechanism against apoptosis. Autophagy should be regarded as a vital protective mechanism, ensuring cell survival in response to multiple types of stress conditions, like hypoxia, elevated levels of ROSs, damaged DNA, or nutrient deprivation [37,38]. Inside the biological systems, apoptosis represents a major form of controlled cell death, a key event in the initiation and development of this process being the caspases’ activation [39,40]. Taken together, we investigated the expression of LC3B, a key autophagy biomarker, and we have also investigated the levels of caspase 3/7 (Figure 7).

Our results illustrated an increment of LC3B levels in the fibroblasts incubated for 24 h with MA-based CAD/CAM samples (Figure 7), suggesting an upregulated autophagic flux. Similar results have been reported by the study of Teti et al. [41], which was focused on investigating the behavior of human gingival cells exposed to 2-hydroxy-ethyl methacrylate (HEMA) and triethylene glycol dimethacrylate (TEGDMA), two free monomers. Their results revealed that HEMA first triggered autophagy, which was then followed by apoptosis. Interestingly, in the case of human gingival cells exposed to TEGDMA, no sign of autophagy activation has been observed. These results indicated that the cells responded to the monomer-induced stress by inducing different adaptive mechanisms in order to restore cellular homeostasis [41]. However, our results revealed no significant differences for caspase 3/7 in the cells incubated for 24 h with CAD/CAM samples versus control. Therefore, these findings could indicate an adaptative, pro-survival feature of autophagy, which is responsible for maintaining the balance between cellular components’ formation and the breakdown of damaged and unnecessary cellular constituents [42,43,44]. However, autophagy should be regarded as a double-edged sword. Up to a certain point, an increase in the autophagic flux may be beneficial by ensuring the elimination of compromised molecules and aged organelles to provide necessary precursors and substrates. In this way, autophagy gives cells the opportunity to adapt and survive in stressful conditions, including exposure to different kinds of dental materials [42,43,45]. Also, the ability of autophagy to downregulate the classical apoptotic pathway by inhibiting caspase activation and clearing damaged mitochondria has been outlined [42,43,45]. Moreover, recent studies pointed out that autophagy inhibited the apoptosis initiation by eliminating the proteins and mitochondria damaged by ROSs under oxidative stress conditions [38,45]. On the other hand, the excessive activation of autophagy might actually trigger cell self-digestion and/or apoptosis [38,45].

Interestingly, the gingival fibroblasts exposed to MA-based 3D samples displayed apoptotic morphological features, including cell shrinking, membrane blebbing, and apoptotic bodies formation (Figure 3), as described previously [32], being in agreement with the LDH release (Figure 4). Furthermore, the 24 h of incubation with MA-based 3D samples triggered a significant increase in LC3B levels (*p* < 0.001) (Figure 7) and caspase 3/7 activity (Figure 6c) compared to the control. Taking into consideration these changes and the significantly reduced cell viability (Figure 2 and Figure 3), it could be suggested that, in the case of MA-3D-24h, autophagy should be regarded as a first step toward apoptosis, rather than an adaptative cellular response. Similar results, reported by Diomede et al. [46], revealed a decreased cell proliferation along with morphological changes in the HEMA-treated dental pulp stem cells. Their findings also revealed that the expression levels of the autophagy-related proteins Beclin-1 and LC3B-I/II were significantly elevated in HEMA-treated cells [46]. One of the key events that trigger apoptosis induction via autophagy signaling might be the selective autophagic degradation of the anti-apoptotic proteins, such as Bruce [45,46,47,48]. Moreover, after stimulating the death receptors, autophagy may also be able to selectively remove the active caspase, delaying the extrinsic apoptosis initiation [45]. Usually, the apoptotic process is illustrated by a high degree of caspase activity [45]. It has been outlined that, in certain circumstances, caspases are able to digest several essential autophagy proteins, including Beclin-1, ATG3, and ATG4, triggering the autophagic flux decrement and, finally, its inhibition, probably in order to abolish its cytoprotective role and to accelerate cellular death [45]. The line between the two roles of autophagy, as a cell survival promoter on one hand, and as a cell death inducer on the other hand, is very delicate and critical, raising questions not answered yet, even in the context of dental material biocompatibility.

Considering that unreacted MA-based monomers are able to induce cellular oxidative stress [49,50], and autophagic machinery is one of the best fighters against this process, our next goal was to explore the possible presence of oxidative stress in the fibroblasts exposed to the two types of dental materials. It is already known that MA-based monomers and co-monomers can induce cellular oxidative stress, most probably by depleting the level of GSH (one of the most important intracellular antioxidants) and, consequently, increasing ROS production [49,50]. Our results revealed a significant decrease in the GSH level compared to control after 24 h of incubation with MA-based 3D-printed samples, which correlated very well with the ROS generation (an increase of 33% above control after the same period of exposure) (Figure 6), suggesting the initiation of oxidative stress. Furthermore, the significantly elevated activity of caspase 3/7 (142% of control) for these 3D-printed samples suggested that the noticed oxidative stress (Figure 6b) might have induced the apoptotic cascade in the gingival fibroblasts. MA monomers’ toxicity has been investigated in several in vitro studies [51,52,53,54]. These monomers have specific chemical properties, due to the carboxylic ester group conjugated with a double bond between alpha- and beta-carbon of the acid moiety, being able to undergo a Michael-type addition to nucleophiles [11]. MA binding to the cysteine residue in GSH has been pointed out as a key event in the reported toxic responses [11], the main consequence of GSH reduction being oxidative stress generation [55,56]. Therefore, the decrease in intracellular GSH levels, possibly as a consequence of adduct formation with MA molecules, may explain the intense oxidative stress observed in the gingival fibroblasts exposed to MA-based samples [55], and it could be considered one of the first steps toward cell death by apoptosis [55], as our findings revealed.

Spagnuolo et al. highlighted that the dental resin methacrylate monomers were able to induce apoptosis in vitro [54]. Cohen et al. have shown that the unreacted MA-based monomers from resin-based dental materials are able to induce cellular oxidative stress by increasing the cellular ROS levels and, consequently, causing the destabilization of the cellular redox balance [39,40]. However, many studies conducted in the recent period highlighted oxidative stress as the converging point of multiple stressful factors, with ROSs taking over the role of main intracellular signal transducers for initiating and sustaining autophagy [57] caused by oxidatively damaged molecules’ accumulation. GSH acts as a direct antioxidant, being, as well, an important substrate for glutathione peroxidase to prevent lipid peroxides’ accumulation. In other words, GSH reduction may ultimately lead to increased lipid peroxidation and ROS accumulation, triggering redox cellular balance dysregulation [55]. ROSs are able to activate autophagy, which will substantially contribute to oxidative stress attenuation by degrading and recycling oxidatively damaged macromolecules and dysfunctional organelles, finally ensuring cell adaptation and homeostasis [38,57]. Wojciech et al. reported that an antioxidant treatment prevented autophagy acceleration, outlining the idea that the redox imbalance had an important role in controlling the autophagic pathway [38]. Therefore, the chemically induced GSH oxidation can trigger autophagy upregulation, even in the absence of any other autophagic inducer. However, due to the little information regarding GSH depletion’s direct effect on autophagy [58,59], it still remains difficult to establish that the increased level of LC3B-I shown by our results in the MA-3D cells represents a direct consequence of MA-induced GSH decrease. Thus, it is essential to further investigate the potential mechanism underlying the GSH depletion–autophagy interplay, especially in the presence of new types of dental materials.

The studies conducted by Chang et al. [60] and Sapna et al. [61] highlighted that MMP-2 might have key roles in collagen turnover during gingival repairing processes and dental splint integration [60,61,62,63,64]. Our results revealed that 24 h of incubation with the MA-based 3D samples triggered no significant differences in the MMP-2 levels. However, a significant increase in the MMP-2 level (*p* < 0.001) compared to control (Figure 8) was observed in the gingival fibroblasts incubated with MA-based CAD/CAM for 24 h. Cell adhesion to dental material surfaces relies on the availability of particular protein-binding sites [65]. MMP-2, the fibroblast-secreted gelatinase, is an important player in the ECM turnover [60,61,62,63,64]. Therefore, our findings might suggest an adaptative tendency of the gingival fibroblasts in the presence of CAD/CAM samples for 24 h. The limitations of our study consist of the fact that we did not perform bacterial adhesion tests, nor did we analyze the effects of the bacterial biofilm on the defining properties for biocompatibility. Another possible limitation of our in vitro study is the use of the MTT viability test, considering the fact that the cells might have had low metabolic activity and instead been illustrated as “dead” by this test.

## 5. Conclusions

Based on our study and previously published data, we can conclude that the different cellular and molecular responses generated during the incubation with the two types of MA-based resin samples might mainly reside in the fabrication method. Our results showed that the MA-CAD/CAM samples displayed good biocompatibility in the presence of human gingival fibroblasts during the 24-h exposure.

Considering that the polymerisation degree is controllable by the monomer-to-polymer ratio, polymerisation reaction temperature, and time, these parameters should be regarded as main targets in order to improve the biocompatibility of the newly-introduced dental resins. Moreover, in the case of photo-polymerization, the curing time and light density should also be taken into account in further biocompatibility studies.

Furthermore, the MA-based 3D-printed samples could be proper for short-term utilizations. More precisely, this MA-based 3D material should be considered for the fabrication of dental splints for provisional restorations, such as surgical guides and oral obturators, strictly following the manufacturer’s protocols. Furthermore, our investigation opens a challenging way to improve all the steps of the 3D-printing technique, especially the curing and washing, for obtaining better biocompatibility.

## Figures and Tables

**Table 1:** Tested materials’ characteristics, manufacturers, and lot numbers.

Tested Sample	Characteristics	Manufacturer	Lot Number
**MA-based** **3D-printed**	Very tough, non-irritant, odourless resin. Colour A1–A2 according to Vita scale.	Dental Sand A1–A2 by HARZ Labs LLC, (Moscow, Russian Federation) Official Dealer: 3D Printing Zone SRL, (Bucharest, Romania)	Dental A1–A2 Lot 107 production date: february 2020
**MA-based CAD/CAM**	Monolayer, shade B1 according to Vita scale. Ultimate flexural strengh ≧ 65 MPa, water absorbtion ≦ 32 μg/mm^3^.	HUGE PMMA BLOCK for CAD/CAM, MedNet EC-REP GmbH (Muenster, Germany)	LOT 200220010, Expiration date: 19 February 2025

## Data Availability

The original contributions presented in the study are included in the article, further inquiries can be directed to the corresponding author.
